# Complete genome sequences of five aquatic *Janthinobacterium lividum* strains collected in Austria

**DOI:** 10.1128/mra.00962-25

**Published:** 2025-11-18

**Authors:** Marie-Therese Fischer, Joana Séneca, Jillian M. Petersen

**Affiliations:** 1 Centre for Microbiology and Environmental Systems Science, University of Vienna 27258, Vienna, Austria; 2 Joint Microbiome Facility, University of Vienna 27258, Vienna, Austria; 3 Department of Laboratory Medicine, Medical University of Vienna 27271, Vienna, Austria; 4 Vienna Doctoral School of Microbiology and Environmental Science, University of Vienna 27258, Vienna, Austria; 5 Environment and Climate Hub, University of Vienna 27258, Vienna, Austria; Montana State University, Bozeman, Montana, USA

**Keywords:** Oxford Nanopore, violacein biosynthesis

## Abstract

We report complete circular genomes of five *Janthinobacterium lividum* strains isolated from Austrian freshwater sources. Genomes (6.3–6.6 Mbp, >99% complete) were assembled using Oxford Nanopore sequencing. Each encodes a full violacein biosynthesis pathway. Phylogenomic analysis places most strains close to the type strains, with one strain more distantly related.

## ANNOUNCEMENT

*Janthinobacterium* (family *Oxalobacteraceae*) is a genus of aerobic, chemoheterotrophic bacteria found in diverse environments (1, 2). A hallmark of many strains is their ability to produce violacein, a purple secondary metabolite known for its attractive color and antimicrobial properties (3, 4). This genus, and particularly the species *J. lividum*, finds use in bacteriographic art (https://publicartists.online/artists/bacteriograph/), pigment production (5), and probiotic interventions (6, 7). Here, we describe five *J. lividum* strains isolated from different environments across Austria (Table 1). Originally selected for artistic contexts, these strains are now being explored for violacein production in interaction with the skin-associated microbiota of native Austrian amphibians. All strains were isolated by spreading 100 µL of water from different sources (Table 1) onto 1% Tryptone Agar plates, evenly distributing the liquid with a sterile spreader, and incubating the inverted plates at 25°C for 1 week (8). Colonies exhibiting a characteristic purple pigmentation were selected for isolation-streaking before proceeding for whole-genome sequencing.

**TABLE 1:** Relevant statistics for sequencing and genome assembly^*a*^

Strain ID	Source/coll. date	Provenance (latitude, longitude)	Read *N*_50_ (bp)	Read # (k)	NP data (Mbp)	# Contigs	Assembly size (bp)	NP_ cov	*N*_50_ (bp)	Largest contig (bp)
WE18	Well 02.04.2023	Vienna (48.235384°N, 16.424592°E)	23,409	21	500	1	6,333,228	80	6,333,228	6,333,228
V02	Well 17.11.2000	Oberpullendorf (47.496927°N, 16.507181°E)	32,685	15	500	1	6,359,883	79	6,359,883	6,359,883
A01	Rain 19.10.2013	Süßenbrunn (48.310280°N, 16.500205°E)	17,552	31	500	1	6,453,713	77	6,453,713	6,453,713
A16	Rain 21.01.2014	Vienna (48.235384°N, 16.424592°E)	14,533	42	500	3	6,579,817	78	6,395,577	6,395,577
J59	Traun river 23.07.2013	Bad Ischl (47.709954°N, 13.621084°E)	13,229	38	500	1	6,422,333	77	6,422,333	6,422,333

Strain ID	Contamin	Completeness	GC (%)	# CDs	Avg protein length	Coding ratio	# rRNAs	# tRNAs	NCBI BioSample ID	Accession # raw reads
WE18	1.38	99.57	62.51	5,543	342	89.8	17	101	SAMN50660558	SRX30138661
V02	1.57	99.57	62.63	5,599	340.4	89.9	16	97	SAMN50660559	SRX30138662
A01	1.97	99.45	62.74	5,674	341.4	90	16	95	SAMN50660560	SRX30138663
A16	2.44	99.65	62.7	5,647	340.1	90.1	16	97	SAMN50660561	SRX30138664
J59	2.04	99.65	62.63	5,696	338.3	90	16	97	SAMN50660562	SRX30138665

^*a*^ DFAST annotations were used to provide number of CD, average protein length, coding ratio, and number of rRNAs and tRNAs. Abbreviations: #, number; CDs, coding DNA sequences; coll. Date, collection date; Cont, contamination; GC (%), GC content; *N*_50_ (bp), contig length at which 50% of the assembly is contained in contigs of this length or longer; NP data (Mbp), Nanopore data yield in megabase pairs; NP_cov, Nanopore coverage; Read N50 (bp), median read length at which half of total bases are in reads of this length or longer.

Strains for sequencing were grown in 10 mL 1% Tryptone media on a rocking platform (100 rpm, 25°C) for 3 days. High molecular weight DNA was extracted using the Monarch gDNA purification kit (NEB), with the specific protocol for Gram-negative bacteria. Samples were size selected using the Short Read Eliminator XS (Pacific Biosciences, USA), and equimolarly barcoded using the Rapid Barcoding Kit 96 (SQK-RBK114.96, Oxford Nanopore Technologies, UK) with the following protocol modifications: sample input was increased to 230 ng, and +0.5 µL of rapid adaptor was added to the barcoded library. About 70 fmol of a 23 Kb library were sequenced on a PromethION (P2 solo, Oxford Nanopore Technologies) for 48 h. Raw signal was basecalled using Dorado (v.0.9.1, https://github.com/nanoporetech/dorado) using model r1041_e82_400bps_sup_v5.0.0). Raw reads were quality filtered with Filtlong (v0.2.1; github.com/rrwick/Filtlong, *Q* ≥ 15, ≥1 kb, 500 Mb retained), assembled with flye (v2.9.5, “–nano-hq,” [9]), manually circularized based on repeat graph inspection, and polished once with Medaka (v2.0.1, “–bacteria,” github.com/nanoporetech/medaka). Contigs smaller than 1,000 bp were removed. Assemblies were quality controlled using QUAST (v.5.3.0 [10], CheckM (v. 1.2.3 [11]) and classified using GTDBtk (v.2.4.0 [12]). Annotation of the largest circular contig was performed using DFAST v1.1.0 from the DDBJ (13). We assessed violacein pathway completeness (KEGG module M00808) (14) using anvi-estimate-metabolism (15), based on KOfam annotations. GTDBtk confirmed the genus assignments as *Janthinobacterium* (domain Bacteria; phylum Pseudomonadota; class Gammaproteobacteria; order Burkholderiales; family *Burkholderiaceae*).

**Fig 1:**
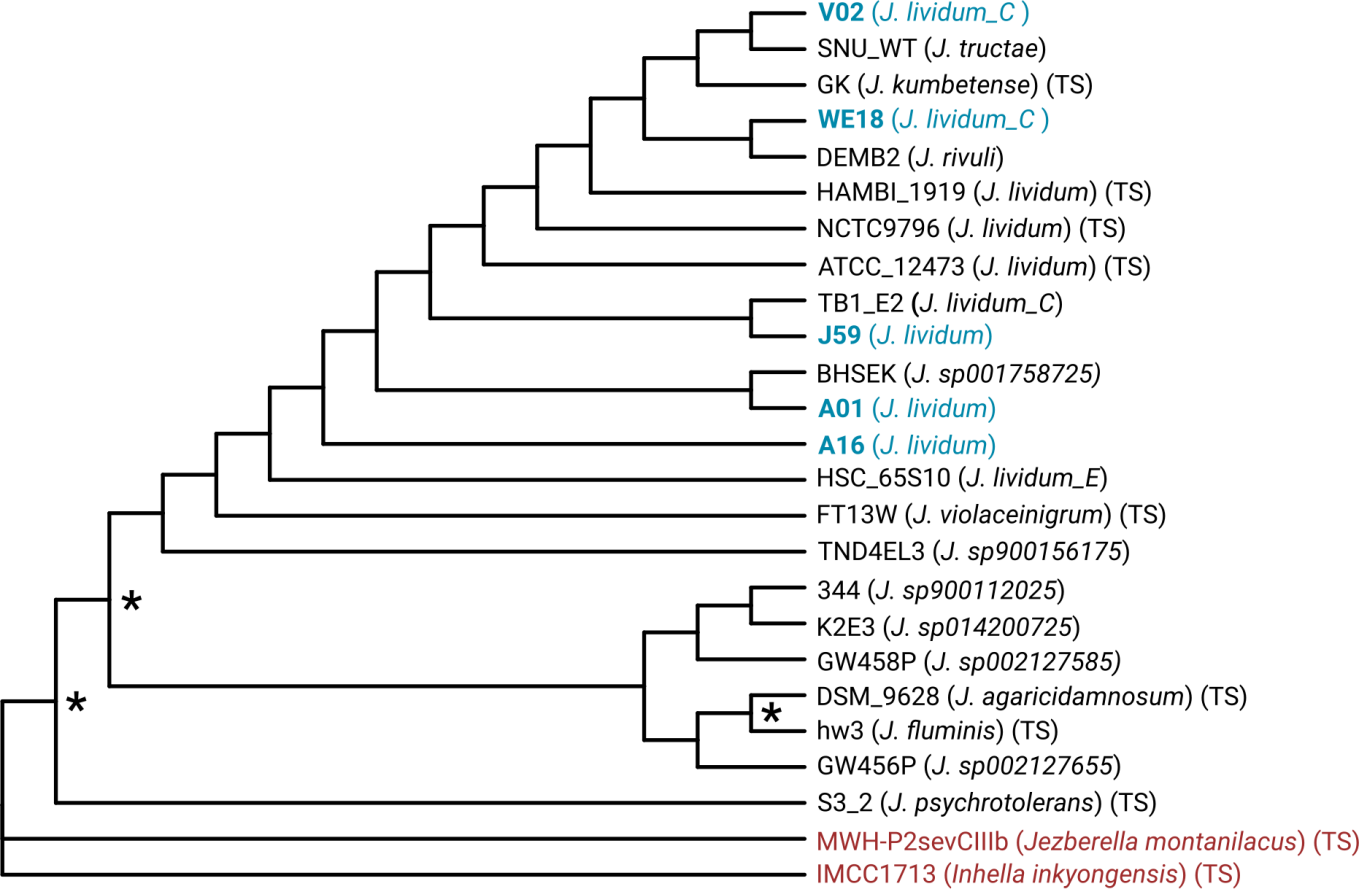
Phylogenomic tree based on concatenated ribosomal protein sequences from *Janthinobacterium* isolates. Taxonomic classifications of the newly described strains were assigned at the species level using GTDB-Tk. Newly sequenced isolates are shown in blue, outgroups in red, and type strains are indicated by (TS). We selected all NCBI-designated Janthinobacterium type strains and GTDB representative genomes of lineages that had complete assemblies available to infer a maximum-likelihood phylogeny with IQ-TREE v2.4.0 (16) from an alignment of 10 concatenated ribosomal-protein sequences (25 taxa and 1,486 amino-acid sites, created with Anvi’o [17]). Using ModelFinder to select the substitution model, the best-fit model by BIC was Q.yeast + F + I + R2. Branch support was evaluated based on 1,000 ultrafast bootstrap replicates and 1,000 replicates of the Shimodaira–Hasegawa approximate likelihood ratio test (SH-aLRT). The final tree was rooted using type strains of the sister genera *Inhella* and *Jezberella* within the *Burkholderiaceae* family as outgroups and visualized in R 4.3.2 (18) with the ggtree package (19). Branches with support >70% are marked with an asterisk (*). The included strains and their assemblies are: *J. lividum* HAMBI_1919 (GCF_034424625.1), ATCC 12473 (GCF_020858175.1), NCTC9796 (GCF_900451145.1), V02 (this study), WE18 (this study), J59 (this study), A01 (this study), and A16 (this study); *J. agaricidamnosum* DSM 9628 (GCF_000275545.1), *J. fluminis* hw3 (GCF_028595325.1); *J. kumbetense* GK (GCF_023703445.1); *J. psychrotolerans* S3-2 (GCF_001677885.1); *J. violaceinigrum* FT13W (GCF_009208555.1); *J. rivuli* DEMB2 (GCF_029690045.1); *J. tructae* SNU_WT1 (GCF_006517255.1); *J. lividum_C* TB1-E2 (GCF_036885605.1); *J. lividum_E* HSC-65S10 (GCF_011601275.1); and *Janthinobacterium* sp. TND4EL3 (GCF_900156175.1), 344 (GCF_900112025.1), BHSEK (GCF_003667705.1), GW458P (GCF_002127585.1), GW456P (GCF_002127655.1), and K2E3 (GCF_014200725.1). Outgroups *Jezberella montanilacus* MWH-P2sevCIIIb (GCF_003003055.1) and *Inhella inkyongensis* IMCC1713 (GCF_005952805.1) were used to root the tree, and branch length was equalized for visualization.

Sequencing and assembly statistics are summarized in Table 1. Phylogenomic analysis (Fig. 1) placed strains J59, A01, and A16 in the core *J. lividum* clade with the three type strains, whereas WE18 and particularly V02 were more distantly related. All five genomes encode a complete violacein biosynthesis pathway, with all enzymatic steps and biosynthetic genes (*vioA–E*) present. These genomes will support future comparisons of strain-level diversity linked to colonization of amphibians versus aquatic environments.

## Data Availability

Sequencing data were deposited in the NCBI Sequence Read Archive (SRA) under a BioProject accession number PRJNA1297442.

## References

[1] De Ley J, Segers P, Gillis M. 1978. Intra- and intergeneric similarities of Chromobacterium and Janthinobacterium ribosomal ribonucleic acid cistrons. Int J Syst Bacteriol 28:154–168. doi:10.1099/00207713-28-2-154

[2] Wu X, Kazakov AE, Gushgari-Doyle S, Yu X, Trotter V, Stuart RK, Chakraborty R. 2021. Comparative genomics reveals insights into induction of violacein biosynthesis and adaptive evolution in Janthinobacterium. Microbiol Spectr 9:e0141421. doi:10.1128/Spectrum.01414-21 34908429 PMC8672880

[3] Brucker RM, Harris RN, Schwantes CR, Gallaher TN, Flaherty DC, Lam BA, Minbiole KPC. 2008. Amphibian chemical defense: antifungal metabolites of the microsymbiont Janthinobacterium lividum on the salamander Plethodon cinereus. J Chem Ecol 34:1422–1429. doi:10.1007/s10886-008-9555-7 18949519

[4] Pantanella F, Berlutti F, Passariello C, Sarli S, Morea C, Schippa S. 2006. Violacein and biofilm production in Janthinobacterium lividum. J Appl Microbiol 0:061120055200056 doi:10.1111/j.1365-2672.2006.03155.x 17381742

[5] Kanelli M, Mandic M, Kalakona M, Vasilakos S, Kekos D, Nikodinovic-Runic J, Topakas E. 2018. Microbial production of violacein and process optimization for dyeing polyamide fabrics with acquired antimicrobial properties. Front Microbiol 9:1495. doi:10.3389/fmicb.2018.01495 30042746 PMC6048185

[6] Becker MH, Brucker RM, Schwantes CR, Harris RN, Minbiole KPC. 2009. The bacterially produced metabolite violacein is associated with survival of amphibians infected with a lethal fungus. Appl Environ Microbiol 75:6635–6638. doi:10.1128/AEM.01294-09 19717627 PMC2772424

[7] Harris R, Lauer A, Simon M, Banning J, Alford R. 2009. Addition of antifungal skin bacteria to salamanders ameliorates the effects of chytridiomycosis. Dis Aquat Org 83:11–16. doi:10.3354/dao02004 19301631

[8] Atlas RM. 2010. Handbook of microbiological media. CRC Press.

[9] Kolmogorov M, Yuan J, Lin Y, Pevzner PA. 2019. Assembly of long, error-prone reads using repeat graphs. Nat Biotechnol 37:540–546. doi:10.1038/s41587-019-0072-8 30936562

[10] Gurevich A, Saveliev V, Vyahhi N, Tesler G. 2013. QUAST: quality assessment tool for genome assemblies. Bioinformatics 29:1072–1075. doi:10.1093/bioinformatics/btt086 23422339 PMC3624806

[11] Parks DH, Imelfort M, Skennerton CT, Hugenholtz P, Tyson GW. 2015. CheckM: assessing the quality of microbial genomes recovered from isolates, single cells, and metagenomes. Genome Res 25:1043–1055. doi:10.1101/gr.186072.114 25977477 PMC4484387

[12] Chaumeil P-A, Mussig AJ, Hugenholtz P, Parks DH. 2020. GTDB-Tk: a toolkit to classify genomes with the Genome Taxonomy Database. Bioinformatics 36:1925–1927. doi:10.1093/bioinformatics/btz848 PMC7703759 31730192

[13] Tanizawa Y, Fujisawa T, Nakamura Y. 2018. DFAST: a flexible prokaryotic genome annotation pipeline for faster genome publication. Bioinformatics 34:1037–1039. doi:10.1093/bioinformatics/btx713 29106469 PMC5860143

[14] Kanehisa M, Furumichi M, Tanabe M, Sato Y, Morishima K. 2017. KEGG: new perspectives on genomes, pathways, diseases and drugs. Nucleic Acids Res 45:D353–D361. doi:10.1093/nar/gkw1092 27899662 PMC5210567

[15] Veseli I, Chen YT, Schechter MS, Vanni C, Fogarty EC, Watson AR, Jabri B, Blekhman R, Willis AD, Yu MK, Fernàndez-Guerra A, Füssel J, Eren AM. 2025. Microbes with higher metabolic independence are enriched in human gut microbiomes under stress. eLife 12:RP89862. doi:10.7554/eLife.89862 40377187 PMC12084026

[16] Minh BQ, Schmidt HA, Chernomor O, Schrempf D, Woodhams MD, von Haeseler A, Lanfear R. 2020. IQ-TREE 2: new models and efficient methods for phylogenetic inference in the genomic era. Mol Biol Evol 37:1530–1534. doi:10.1093/molbev/msaa015 32011700 PMC7182206

[17] Eren AM, Kiefl E, Shaiber A, Veseli I, Miller SE, Schechter MS, Fink I, Pan JN, Yousef M, Fogarty EC, et al.. 2021. Community-led, integrated, reproducible multi-omics with anvi’o. Nat Microbiol 6:3–6. doi:10.1038/s41564-020-00834-3 33349678 PMC8116326

[18] R Core Team. 2023. R: a language and environment for statistical computing. R Foundation for Statistical Computing, Vienna, Austria.

[19] Xu S, Li L, Luo X, Chen M, Tang W, Zhan L, Dai Z, Lam TT, Guan Y, Yu G. 2022. Ggtree: a serialized data object for visualization of a phylogenetic tree and annotation data. Imeta 1:e56. doi:10.1002/imt2.56 38867905 PMC10989815

